# Refined utilitarianism in practice: reinterpreting the ethical foundations of the NHS

**DOI:** 10.1186/s13010-025-00206-x

**Published:** 2026-02-05

**Authors:** Ahmet Küçükuncular

**Affiliations:** Near East University, Nicosia (Lefkoşa), Turkey

**Keywords:** NHS, United Kingdom, COVID‑19, Refined utilitarianism, Healthcare resource allocation, QALY, Cost‑effectiveness analysis, Triage, Healthcare ethics, NICE (National Institute for Health and Care Excellence)

## Abstract

The COVID-19 pandemic exposed acute tensions in the United Kingdom’s National Health Service (NHS) between its egalitarian self-image and the utilisation of overtly utilitarian tools such as QALY-based cost-effectiveness and prognosis-driven triage. This paper offers a systematic philosophical diagnosis of those tensions through the lens of *refined utilitarianism*, a consequentialist theory that grounds moral rules in their long-run welfare effects while requiring genuine social approbation and cultural fit. After reconstructing the classical act-/rule-utilitarian debate and engaging canonical critics (Rawls, Scanlon, Dworkin and Williams), the paper distinguishes refined utilitarianism from both rule utilitarianism and prioritarianism, then tests the theory against UK healthcare practice pre- and post-pandemic. Using documentary analysis of NICE technology appraisals, BMA emergency guidance, and statutory equality duties, it is shown that the NHS operates a two-tier code: egalitarian, need-based rules in conditions of routine capacity, and outcome-maximising rules, tempered by fairness safeguards, during acute scarcity. This structure closely matches refined utilitarian predictions: apparently non-utilitarian norms (equal access, prohibition of direct age discrimination, ‘rule of rescue’) are retained because they enhance trust, compliance and therefore aggregate welfare, while harsher maximisation moves (frailty scoring, ICU reallocation, staff testing priority) are publicly justified and time-limited to preserve legitimacy. The analysis answers familiar objections, impracticality of hedonic calculus, neglect of minorities, integrity threats, by showing how socially embedded rules mitigate each problem without surrendering the aim of greatest overall benefit. The paper concludes that UK healthcare is best interpreted as *utilitarian in a refined sense,* and that refined utilitarianism provides a practicable, normatively attractive template for future resource-allocation frameworks in public health emergencies and beyond.

## Clinical trial number

Not applicable.

## Introduction

The COVID-19 pandemic has thrust moral philosophy into the spotlight of public policy, especially in healthcare. At the peak of the crisis, dire shortages of critical resources (such as ventilators, intensive care beds, and staff) forced healthcare systems to confront hard ethical choices. Notably, the British Medical Association (BMA) issued emergency guidance in 2020 urging a shift “*towards the utilitarian objective of equitable concern for all – while maintaining respect for all as ‘ends in themselves*” [1, p. 4]. In other words, during a dangerous pandemic, doctors should focus on saving as many lives as possible (a utilitarian stance), even as they continue to treat individuals with dignity. This official endorsement of a more utilitarian approach to triage provoked debate about the moral underpinnings of the National Health Service (NHS). Is the NHS, ordinarily celebrated for its egalitarian ethos of equal access based on need, fundamentally utilitarian when allocating healthcare? Or was the pandemic a rare departure from an otherwise non-utilitarian framework? These questions invite a deeper examination of how UK healthcare balances utilitarian goals (such as maximising health outcomes) with egalitarian commitments (such as treating patients equally and respecting individual rights). Rather than treating COVID‑19 as an ethical turning point, I treat it as a stress test that made explicit a two‑tier structure already implicit in NHS rules (egalitarian/need‑based norms in routine conditions, outcome‑maximising rules in acute scarcity). This reframing, relatedly, sets up a normative inquiry: what framework best justifies that structure?

In this article I develop and defend refined utilitarianism for healthcare priority‑setting. My secondary aim is interpretive, and is to show that many NHS rules – before and during COVID‑19 – fit refined utilitarianism’s predictions. Refined utilitarianism is a specific interpretation of utilitarian theory that recognises the importance of rules, social values, and context in producing the greatest overall good. In brief, it holds that actions should be guided by rules whose general acceptance maximises long-term welfare, even if following those rules in particular cases does not yield the highest immediate utility. In other words, refined utilitarianism adds three features that distinguish it from standard rule utilitarianism: (i) a deliberative acceptance requirement (endorsement under transparent, non‑manipulative, well‑informed conditions), (ii) explicit context codes (routine vs crisis), and (iii) a rights‑protecting baseline of prohibitions that any sustainable public code must entrench. In healthcare, refined utilitarianism illuminates why some practices are overtly utilitarian (for example, allocating scarce organs or ICU beds to those likely to benefit most), whereas other practices appear to reflect egalitarian or rights-based principles (for example, the NHS commitment to equal treatment and the legal prohibition on discrimination). The central claim is that even the seemingly non-utilitarian aspects of NHS practice can serve a utilitarian aim once one ‘looks at the bigger picture’ of societal impact and public trust. By accounting for the difference between the *immediate* utility of specific acts and the *overall* utility of following certain rules, refined utilitarianism bridges the gap between utilitarian reasoning and values like fairness and equity. In doing so, it offers a nuanced ethical framework that both explains the status quo and guides how healthcare resources ought to be distributed.

To develop this argument, I begin by reviewing the foundations of utilitarian ethical theory and its classic criticisms. I then introduce refined utilitarianism and distinguish it from other approaches, particularly *rule utilitarianism* and *prioritarianism*.

After establishing this theoretical framework, I examine what a purely utilitarian healthcare system would entail, for example, use of cost-benefit analyses like Quality-Adjusted Life Years (QALYs) to allocate resources, and what challenges such a system faces. Next, I consider the current NHS principles and practices: on the surface, UK healthcare espouses egalitarian ideals of equal access and prioritising the sickest, yet it also employs utilitarian-informed tools for rationing and triage. One shall see that in normal conditions the NHS follows rules that ensure equity and inclusivity (which refined utilitarianism endorses for their long-term benefits), whereas in emergencies or resource scarcities, more direct utilitarian criteria come to the fore. Throughout, I engage with seminal philosophers – including John Rawls, T. M. Scanlon, Ronald Dworkin, and Bernard Williams – who have been critical of utilitarianism, to clarify how refined utilitarianism addresses concerns about justice, rights, and individual integrity. Real policy examples (such as UK COVID-19 triage protocols and NICE’s use of QALY thresholds for funding decisions) are used to illustrate the theory in practice, with reference to authoritative sources (NHS, NICE, BMA, UK law). In sum, with this article, I aim to show that UK healthcare can be best interpreted as *utilitarian in a refined sense*: it seeks to maximise overall health and well-being, not through crude calculus at the bedside, but via a system of rules and institutions shaped by societal values and empirical experience. Such an approach, I argue, retains much of utilitarianism’s aggregate welfare orientation while incorporating important correctives to its harsher implications, thereby offering a morally and practically sound guiding philosophy for healthcare.

## Utilitarianism: classical foundations and criticisms

Utilitarianism is one of the major normative ethical theories in Western philosophy. At its core is the principle of utility, often summarised as doing the greatest good for the greatest number. Classical utilitarianism emerged with the work of Jeremy Bentham (1748–1832), who defined utility as whatever promotes pleasure or happiness and prevents pain or suffering [2]. Bentham proposed that one can quantify pleasures and pains using a *hedonic calculus*, assigning values to an action’s likely consequences for everyone affected, and thereby determine morally right actions by comparing the net happiness produced. As John Stuart Mill (1806–1873) later phrased it, actions are right in proportion to the extent they promote happiness (pleasure and the absence of pain), and wrong as they produce the reverse of happiness [3, p. 6]. In Bentham’s formulation, each individual’s happiness counts equally, and one simply sums up positive and negative utilities; the morally best choice is that which yields the highest total balance of benefit over harm for all involved. This commitment to impartiality and aggregate welfare was revolutionary in Bentham’s time. It had a progressive, egalitarian spirit: everyone’s well-being mattered, even those of the disenfranchised, and laws or actions were to be judged by their contribution to human flourishing [4, 5]. Utilitarian reformers like Bentham and Mill thus challenged elitist or tradition-bound policies by asking whether they actually benefited people – if a law caused more harm than good, it should be changed [2, 4]. In this sense, classical utilitarianism was anti-authoritarian and aimed at the welfare of the many, which resonates with the egalitarian ethos that “*each counts for one, and none for more than one*” (to use a phrase often attributed to Bentham’s follower John Stuart Mill). In what follows I use “utility” in the broader welfare sense (well‑being, including health and the social goods that sustain it), treating classical hedonism as historically important but not exclusive. Also, unless otherwise noted, I assume a total (rather than average) welfare standard, as is customary in public‑policy discussions.

However, the simplicity of act utilitarianism, ie evaluating each action by its direct consequences for overall happiness, quickly invites serious objections. Critics note that if only outcomes matter, a strict act-utilitarian might sanction intuitively unjust or immoral acts in particular situations, so long as those acts maximise net utility. For example, if telling a lie or evading taxes in a given case would produce more happiness overall, act utilitarianism appears to say that one not only *may* but *should* do so. Likewise, in notorious thought experiments, an act-utilitarian calculus could condone sacrificing one innocent person to save five lives, or harvesting the organs of an unwilling healthy patient to save several others, if those actions really did maximise total well-being (a possibility I later show refined utilitarianism categorically forbids at the rule level). Such implications clash with common moral intuitions about justice, rights, and individual dignity. As John Rawls [6] famously argued, utilitarianism does not take seriously the distinction between persons – it is as if the theory regards society as a single super-person and is willing to trade off one person’s welfare for another’s as freely as one might balance benefits and harms within one life. This *separateness of persons* critique underscores that individuals have rights and interests that should not be summarily overridden for aggregate gains (ibid.); this critique presses any plausible utilitarian view to locate evaluation at the level of publicly sustainable rules rather than unconstrained case-by-case maximisation. Similarly, the philosopher T. M. Scanlon [7, 8] contends that morality is about what we owe to each other as individuals and that an action is wrong if it cannot be justified to each person affected, a standard that pure cost-benefit aggregation may fail. From a contractualist perspective (like Scanlon’s), it would never be acceptable to harm or neglect the few solely for the sake of greater good to others if those few could reasonably reject the principle permitting such sacrifice [8]. In rights-based theories, such as Ronald Dworkin’s [9], individuals possess inviolable rights that function as trumps against collective welfare calculations. For example, a hospital cannot deliberately kill one patient to distribute their organs to five others, because that would violate the one patient’s fundamental right to life and equal respect (ibid.). These critiques highlight a tension between utilitarianism and the constraints of justice and rights. Bernard Williams [10] added a further criticism: utilitarianism’s demand for impartial maximisation of welfare can alienate individuals from their personal commitments and moral identity. In Williams’ view, utilitarian reasoning treats the moral agent as a mere channel or conduit for the sum of everyone’s interests, denying the agent’s own projects and integrity [11]. For instance, if a pacifist chemist is told to work on chemical weapons because it will prevent someone more zealous from doing so (thus marginally reducing harm overall), a strict utilitarian rationale would urge him to take the job. But Williams argues this divorces the chemist from his core values, effectively undermining his integrity, by making him responsible for outcomes produced by others if he refuses [10, 11]. In another famous scenario, Jim and the Indians, Williams imagines an explorer asked to execute one innocent person to save 19 others from a ruthless captain. Utilitarianism says Jim *ought* to kill the one to save the many, but Williams points out that this ignores Jim’s personal moral conviction (perhaps abhorrence of killing) and treats his moral feelings as mere data for calculation rather than constraints in themselves [10]. In short, critics like Williams maintain that utilitarianism, by focusing only on outcomes, fails to account for the integrity of moral agents and the intrinsic moral significance of how outcomes are brought about (such as who is responsible for a harm, or whether someone’s rights were violated).

Utilitarians have developed several responses to these challenges. A pivotal move (initiated by J.S. Mill and others) is to distinguish between evaluating actions individually and evaluating rules or general practices. Rule utilitarianism proposes that instead of calculating utility case by case, one should adopt those rules of conduct which, if followed generally, tend to produce the most overall good [3, 12]. In Mill’s words, it would be “*unworthy of an intelligent agent”* not to realise that certain actions belong to *“a class which, if practiced generally, would be generally injurious”*, and that this is why we have an obligation to abstain from them [3, p. 19]. For example, even if one instance of tax evasion or one lie might appear beneficial, a rule-utilitarian argues that one ought not to lie or cheat *as a rule* because widespread lying or tax cheating would undermine social trust, public services, etc., ultimately reducing overall welfare. Thus, a rule against dishonesty or tax evasion is justified by the long-term utility of honest practices. Rule utilitarianism thereby places an indirect check on the ‘ends justify the means’ tendency of act utilitarianism. It forbids certain act-types (like lying, stealing, or killing the innocent) *not* because such acts are intrinsically wrong regardless of consequences, but because a society that permits them would, in the long run, be worse off in terms of overall happiness. In essence, rule utilitarianism considers the wider societal implications and precedent effects of actions: it asks, “What if everyone did this?” [13]. A canonical contemporary development is of 14, which selects the code whose general acceptance would have the best consequences; the point is not to deny consequences but to locate evaluative focus on publicly knowable rules rather than one‑off acts. This shift to rules answers some criticisms of act utilitarianism. Notably, it better protects minorities and individual rights. If following a rule of justice (e.g. *“do not punish an innocent person”*) produces greater trust and security in society, then even a utility-focused view will uphold that rule rather than break it for a one-time gain. Mill himself, in his social and political writings, emphasised the importance of individual liberties and minority rights, which he believed were compatible with utilitarianism because respecting such rights generally promotes the greatest happiness in the civilised community [15]. After all, Mill’s commitment to the equal value of all individuals and to protecting minorities sits more comfortably with rule utilitarianism than with act utilitarianism. The rule-based approach also addresses practical concerns: it is not feasible for people to perform a complex utility calculus for every decision in real time, especially in crises. Secondary principles or rules, such as “prioritise the worst injured in an emergency” or “keep your promises”, serve as *“landmarks and direction-posts”* [3, p. 28] that guide action without constant calculation. These rules are justified because over generations we have learned which kinds of actions tend to promote general welfare [16]. In summary, *act utilitarianism* judges each act by its immediate consequences, whereas *rule utilitarianism* evaluates actions by whether they accord with rules that maximise welfare in the long run.

It is important to note, however, that even rule utilitarianism remains, at its foundation, a consequentialist theory. The ultimate justification for rules is still that they produce good outcomes overall; the difference is just the time horizon and level of generality at which utility is assessed. Philosophers have long debated whether rule utilitarianism collapses into act utilitarianism (since any *particular* case where breaking the rule would yield better results might tempt a fine-tuned exception) or whether it can stably incorporate genuine constraints [17, p. 943]. In practice, most utilitarians endorse some form of *multi-level* thinking: follow general ethical rules and intuitions in day-to-day life (because these normally promote welfare), but be prepared to reconsider them in rare cases or policy-level decisions if following a rule would clearly be disastrous [18, 19]. This two-level utilitarian strategy – intuitive rules for ordinary contexts, critical utility calculations for extraordinary circumstances – is actually quite pertinent to healthcare, as we shall see. It anticipates the idea behind the BMA’s guidance: our everyday medical ethics can prioritise individual needs and equal respect, but in a catastrophe, we might invoke a higher-level principle of saving the most lives [1]. Previewing the view defended below, refined utilitarianism institutionalises this two‑level thought by distinguishing routine and crisis codes, and by adding a public‑acceptability condition to rule selection

Before moving on, one must briefly revisit the objections from Rawls, Scanlon, Dworkin, and Williams in light of these refinements. Rule utilitarianism partially mitigates Rawls’s concern about sacrificing individuals, because well-chosen rules (e.g. “do not convict an innocent person even if it would calm a mob”) will usually prevent gross injustice. Yet Rawls would likely argue this is insufficient, the utilitarian’s commitment to equality is contingent on it maximising welfare, whereas for Rawls justice involves inviolable principles (like equal basic liberties and fair opportunity) that are lexically prior to aggregate welfare [6]. Strictly speaking, Rawls is not a prioritarian, though the difference principle gives lexical priority to improving the position of the worst‑off; we will return to this point when comparing utilitarian and egalitarian healthcare [20]. A Scanlonian contractualist might acknowledge that if a rule is justified because no one could reasonably reject it, then in practice that may align with a rule that maximises long-term utility [8]. For instance, a rule barring doctors from removing one patient’s organs to save others is one no patient could reasonably reject, and it also likely has good consequences (people trust hospitals). However, the crucial difference is one of justification: refined utilitarianism would forbid organ sacrifice because of its bad consequences (e.g. fear, reduced healthcare utilisation), whereas Scanlon forbids it because it cannot be justified to the person used as a means, regardless of consequences. Refined utilitarianism, while gentler than crude utilitarianism, still ultimately justifies moral rules instrumentally by overall welfare. A contractualist or deontologist might worry that if the calculus of overall welfare shifted, the individual could again be at risk, whereas in their view certain individual rights or duties hold categorically. Ronald Dworkin argued that government must treat each person with equal concern and respect, and individuals have rights that trump mere calculations of social utility [9]. A refined utilitarian healthcare system would say it *does* treat people with equal concern – indeed, counting everyone’s happiness equally is a kind of equal concern. But Dworkin would respond that genuine equal concern might mean, for example, not denying treatment to someone just because it is not cost-effective, since that person has a right not to be treated as expendable [21]. Refined utilitarianism tries to accommodate this by generally forbidding discrimination and ensuring a baseline of care for all (because that strategy yields public trust and cooperation). Yet in extreme scenarios (like allocating a single liver to two patients), refined utilitarianism will still favour the patient who yields better outcomes, effectively valuing that life-year more in that context. Dworkin might consider that a violation of the ideal of equal respect, whereas the refined utilitarian defends it as a hard choice required to maximise overall health benefit. We will see this tension in debates on Quality-Adjusted Life Years (QALYs) and whether they unjustly disadvantage people with disabilities or the elderly. Relatedly, Williams’ critique of integrity would imply that rule-based and indirect utilitarian strategies reduce the frequency of morally outrageous demands on individuals. A doctor in normal times can abide by established ethical rules (like treating patients in order of need, or never actively causing a patient’s death) without constantly second-guessing if utility would be higher by doing otherwise. This allows the doctor to preserve personal moral integrity and public trust. However, Williams might argue that the philosophical rationale still does not give intrinsic weight to the doctor’s commitments; it is simply that respecting commitments tends to produce better outcomes [10]. In crisis situations, a refined utilitarian approach might still require deeply distressing actions (like withdrawing treatment from one patient to save another with better prognosis). Indeed, the BMA’s pandemic guidance explicitly countenanced withdrawing ventilators from patients with poorer chances in order to “*deliver the greatest medical benefit to the greatest number*” [1, p. 6]. A clinician forced to carry out that policy might suffer anguish and guilt, a blow to integrity, although a refined utilitarian would say the rule permitting such reallocations is justified by public health emergency needs and, importantly, must be publicly defensible under transparent, non‑manipulative processes. As argued below, refined utilitarianism embeds rights‑protecting baseline rules that categorically bar rights‑violating practices (such as non‑consensual organ procurement), not merely for public‑relations reasons but because any publicly sustainable code permitting them would predictably destroy trust, depress care‑seeking, and thereby reduce welfare over time. In effect, refined utilitarianism attempts to answer Williams by incorporating *negative* side-effects of utilitarian choices (guilt, loss of trust, psychological trauma) into the calculus as costs to be avoided. By generally following commonsense moral rules, refined utilitarianism aligns with what our moral conscience and cultural norms endorse, thereby avoiding many of the intolerable prescriptions that pure act-utilitarianism might generate [22]. Still, the philosophical debate remains open whether this fully addresses Williams’ point or merely postpones it to the rare cases where even our evolved rules demand grave sacrifices.

In summary, classical utilitarianism provides a powerful but oversimplified moral calculus. It is *agent-neutral* and aggregative, treating everyone’s good equally – an outlook well-suited to public policy aims like improving population health. Yet taken to its act-by-act extreme, it clashes with principles of fairness, rights, and personal integrity that many consider essential to ethics. *Refined utilitarianism* emerges as an evolution of the theory, attempting to preserve its strengths (impartial concern for overall well-being) while acknowledging these other moral considerations. By shifting the focus from individual actions to rules and long-term outcomes (as Mill did) and by broadening the notion of utility to include societal values (e.g. the utility of having a just and trusted healthcare system), refined utilitarianism sets the stage for a more sophisticated analysis of healthcare ethics. The remainder of the paper formalises this refinement: I state refined utilitarianism as a decision procedure with a public‑acceptability condition and a rights‑protecting baseline, distinguish it from rule utilitarianism and prioritarianism, and later identify discriminators that mark policies as distinctively utilitarian at the systems level. In the next section, this refined utilitarian approach is more formally defined and distinguished from related concepts like rule utilitarianism and prioritarianism. I shall then apply it to examine how a utilitarian might design a healthcare system, before comparing that model to the actual NHS.

## Utilitarianism refined: rules, context, and social welfare

Refined utilitarianism can be described as a rule-based, context-sensitive form of utilitarian philosophy that explicitly accounts for social approval and long-term consequences in judging what is right. In refined utilitarianism, the moral rightness of an action is determined not by that action’s immediate outcome alone, but by whether it accords with a rule (or policy) that, if generally followed and publicly accepted, would maximise overall welfare. This approach builds on rule utilitarianism but adds an important emphasis: the *social acceptance* of rules and the *broad notion of welfare* that includes societal trust, stability, and other indirect effects. In healthcare ethics, refined utilitarianism means that clinicians and policymakers should follow principles that enjoy public support and promote community values, *provided* those principles lead to the best aggregate outcomes in the long run. Notably, refined utilitarianism permits that in unusual circumstances (such as disasters) a different set of rules may be appropriate than in normal circumstances, reflecting the idea that what maximises welfare can depend on context. In what follows, I make this precise by introducing a decision procedure for refined utilitarianism and by specifying how acceptance is to be understood (as deliberative acceptance under transparent, non‑manipulative, well‑informed conditions) (see Table 1).

**Table 1:** Refined utilitarianism in a nutshell

Refined Utilitarianism: a decision procedure for institutions	
Objective	Choose rules/codes $\mathcal{R}$ that, if publicly adopted and applied over time, maximise population welfare $\mathcal{W}$ (broadly construed: health outcomes, trust, legitimacy, psychological harms/benefits).
Deliberative acceptance	A rule is admissible only if it could be endorsed under transparent, non‑manipulative, well‑informed public deliberation; manufactured consent does *not* qualify.
Context codes	Adopt distinct routine and crisis codes, since $\mathcal{W}$ is context‑sensitive and the same rule need not be optimal across contexts.
Rights‑protecting baseline	Entrench robust prohibitions (e.g. direct discrimination, non‑consensual organ procurement). Refined utilitarianism selects such prohibitions *categorically* because any public code that permits them predictably destroys trust and depresses welfare.
Tie‑breakers & exceptions	When admissible rules are close in $\mathcal{W}$, prefer those that better align with widely shared fairness intuitions (e.g. lotteries for equal claims); craft narrow, sunsetted exceptions where transparency increases.

NB: Refined utilitarianism remains consequentialist but differs from standard rule utilitarianism by building in deliberative acceptance and explicit context codes; it often converges with prioritarian prescriptions instrumentally (not intrinsically)

To clarify, it is useful to distinguish refined utilitarianism from two adjacent ideas: rule utilitarianism and prioritarianism. At first glance, refined utilitarianism sounds very much like standard rule utilitarianism, and indeed it shares the rule-utilitarian insight that one should set one’s compass by general rules rather than ad-hoc calculations [22, 23]. The key difference lies in *how the rules are selected and applied*. Traditional rule utilitarianism typically considers an idealised scenario where a set of moral rules (for example, a code of medical ethics) is internalised or followed by everyone, and it asks which set of rules would yield the highest total utility [14]. It sometimes assumes that the rules are absolute (no breaking them, since they are optimal in general). Refined utilitarianism, by contrast, is more *flexible* and attuned to actual human attitudes. It recognises that the utility of a rule depends on society’s response to that rule. A rule that is too strict or too contrary to prevalent moral instincts might not be effectively followed or could generate backlash, thus undermining utility. Refined utilitarians therefore take into consideration the anthropological and cultural factors influencing moral approbation. That is to say, refined utilitarianism takes notice of the societal involvement in not only preferring one rule to another, but also in what constitutes overall welfare. For example, a purely utility-maximising hospital policy might say: *treat younger patients before older ones in all cases, because it yields more total life-years*. However, if such an ageist rule would cause public outrage, fear among the elderly, or loss of solidarity (thus reducing overall welfare in society), a refined utilitarian would *not* endorse it. Instead, refined utilitarianism might support a more subtle rule (e.g. use clinical prognosis that incidentally favours the young, but do not openly discriminate by age, and maintain a norm of equal respect) that society finds acceptable and that in practice still allocates resources efficiently. Standard rule utilitarianism could in theory reach a similar conclusion, but refined utilitarianism explicitly builds in the requirement of social acceptability as part of evaluating utility. It thereby captures the spirit of John Stuart Mill’s observation that secondary principles (“*do not discriminate by age*” for instance) serve as guides toward the ultimate utilitarian aim (i.e. happiness), much like road-signs help a traveller reach their destination [3, p. 24]. The destination (i.e. maximal welfare) remains, but the route must be navigable by real moral agents in a real society. Crucially, by requiring deliberative (not engineered) acceptance and by recognising explicit context codes and a rights‑protecting baseline, refined utilitarianism avoids the charge of being ad hoc “utilitarianism whenever convenient”.^2^

Another difference in application is that refined utilitarianism countenances context-dependent rules. I hinted that what maximises utility in normal times [when resources are adequate) may differ from what maximises utility in emergency conditions. This view is reminiscent of R. M. Hare’s [18], two-level utilitarianism, wherein one usually adheres to intuitive moral rules but switches to critical calculus in extreme situations. Refined utilitarianism formalises this within a rule framework: it can endorse one set of rules for ordinary healthcare delivery and a modified set for crisis triage. The BMA’s guidance can be interpreted in this light; they did not necessarily claim that the principles of NHS care (such as compassion and respect for persons] change in a pandemic, but they did recommend that the rules of allocation shift to a more utilitarian mode (prioritising those likeliest to benefit) when resources are overwhelmed [1, 24]. Refined utilitarians justify this by noting that in catastrophic scenarios, the public’s expectations also shift: people generally accept that saving more lives is the overriding goal, *as long as* a basic standard of care and transparency is maintained [25]. In everyday conditions, however, a strictly utilitarian allocation (for example, always favouring patients with higher recovery odds) would offend common morality and might erode trust in the health system. Thus, refined utilitarianism gives different imperatives for different circumstances, guided by what rule people are willing to endorse in each context. This makes it a pragmatic ethical approach: it seeks the best outcomes *within* the constraints of human psychology, social stability, and legal norms, rather than in abstract isolation.

Now let us distinguish refined utilitarianism from prioritarianism, since the former often converges instrumentally with giving some priority to the worse‑off but does not treat such priority as intrinsically required (see Table 1). Prioritarianism (also called the Priority View) is a doctrine in distributive ethics which holds that benefiting people matters more the worse off those people are, morally speaking, even if the gain is the same [21, 26]. Prioritarians agree with utilitarians that one should seek to improve welfare, but disagree that a given benefit has equal value no matter who receives it. Instead, helping someone in great need (or with very low welfare) is morally more urgent than helping someone who is already well off, *even if the amount of increase in welfare is identical* [19]. In formal terms, prioritarianism assigns a weighted value to gains in utility that is higher for individuals at lower baseline welfare [26, 27]. This avoids one of strict egalitarianism’s pitfalls, the levelling down objection, which notes it would be absurd to *reduce* everyone’s welfare to the same low level in the name of equality (e.g. making the healthy as ill as the sick achieves equality but helps no one). Prioritarianism does not value equality for its own sake; it values improving the condition of the worst-off, which often will reduce inequality as a side-effect [26]. In healthcare, a pure prioritarian might say that saving the life of a severely ill patient has greater moral weight than slightly extending the life of a healthier patient, even if by the numbers the life-years gained are the same, because the very ill patient is worse off [28]. Prioritarianism thus favours directing resources to those in greatest need or with the poorest health prospects, *other things equal*, rather than simply maximising total QALYs.

How does refined utilitarianism compare? Interestingly, refined utilitarianism often converges with prioritarian conclusions in practice, but for a different underlying reason. A refined utilitarian can agree that it is usually right to treat the worse-off or sicker patient first because doing so tends to maximise long-run social utility. If people perceive the health system as cruelly abandoning the sick or disabled because they are ‘inefficient’ to treat, public trust and the sense of social solidarity will plummet, outcomes very detrimental to overall welfare. Conversely, a policy that gives some priority to those worst-off (such as emergency triage based on urgency of need) fosters community values like compassion and equality, which have positive utility. Indeed, empirical evidence shows that many people are willing to sacrifice some aggregate health gain in order to prioritise the worse-off in healthcare allocation [29]. In refined utilitarian terms, this means a rule that incorporates prioritarian preference is likely to be more socially accepted and thus yield higher overall satisfaction. For example, consider a scenario with two patients in an emergency room: one is moderately ill, and the other is critically ill (far worse off). A strict utilitarian might note that treating the critical patient yields, say, 5 QALYs of benefit whereas treating the moderate patient yields 6 QALYs (perhaps because the critical patient might not fully recover). Pure utility would then favour the moderate patient. But most people (including doctors) would feel it is right to attend to the critically ill patient first, a prioritarian inclination.

A refined utilitarian would endorse *giving some priority* to the worse-off where it is embedded in a rule that maximises long term welfare (trust, compliance, marginal benefit), but would *not* imply an unconditional “worse-off first” principle. This aligns with the NHS’s ethos of need‑based care and with professional ethics of compassion, and it prevents greater tragedy by averting deterioration among those in dire need [30]. Additionally, the friend or family of the healthier patient is likely to agree that the critical case should take priority, because we all share an intuition to rescue those nearest to death or in greatest peril. In the earlier example, the ambulatory friend is likely not only to allow but to approve of the doctor treating their paraplegic friend first. This general approbation contributes to societal utility, there is no sense of injustice, and everyone trusts that if they were worse-off, they would get priority as well. Thus, refined utilitarianism can justify prioritising the worse-off instrumentally: such prioritisation, when built into a rule, produces better overall outcomes (including intangible goods like social cohesion).

The crucial difference from prioritarianism proper is that a refined utilitarian would not attach intrinsic moral weight to benefiting the worse-off regardless of overall outcomes. If helping a worse-off patient yielded markedly less total benefit and if doing so undermined other patients’ welfare with no countervailing social benefits, the refined utilitarian might not prioritise the worse-off in that case. Prioritarianism *would*, by definition, still favour the worse-off to some extent even if no one else benefits. For instance, pure prioritarianism might say that there is some moral reason to extend a dying patient’s life by one day rather than give a healthy person a year of perfect health, if that dying patient is vastly worse off, though likely it would still choose the larger benefit in practice once weighted, depending on the priority function.^3^ Refined utilitarianism, by contrast, always keeps an eye on the sum of benefits (adjusted for societal impact). It only coincides with prioritarianism to the extent that doing so maximises long-term welfare. Fortunately, in many healthcare situations, helping the worst-off does have high marginal utility: for example, treating a life-threatening injury (worst-off patient) yields a larger gain in total welfare than marginally improving someone’s already decent health. Also, public support strongly favours a rule of rescue, the imperative to save those in immediate peril first, which refined utilitarians consider in their utility calculations [5]. The outcome is, one hereby argues, refined utilitarianism is congruous with the full prioritarian account more so than an act-by-act utilitarianism would be. Where they diverge is in hypothetical cases where overall good and priority conflict: prioritarianism says even if the total good gained is the same, it is morally better if it goes to the worse-off individual [26, p. 213]. An ‘unrefined’ utilitarian would be indifferent in such a case, caring only about the sum. Refined utilitarianism, however, can side with the prioritarian view by observing that a rule favouring the worse-off is more socially acceptable and thus promotes aggregate utility in indirect ways. For example, even if two treatment options produce the same total health benefit, a policy that allocates it to the patient who would otherwise remain worse-off will be seen as fair and will enhance public confidence (a utility gain), whereas the opposite policy could breed resentment (a utility loss). In technical terms, refined utilitarianism acknowledges a kind of diminishing marginal utility of health: improving the health of someone who is very sick adds more to social welfare (in terms of compassion satisfaction, etc.) than an equal improvement to someone already in good health. This parallels the prioritarian idea without committing to it as a fundamental moral axiom. It is a contingent, empirical claim about human values that utilitarians take seriously in policy design [29]. Refined utilitarianism thus *explains* when and why prioritarian‑looking rules are justified *within* a consequentialist framework, without treating priority as lexically dominant.

To illustrate, consider a simple case. Two patients need a kidney transplant: Patient A is young and otherwise healthy (high life expectancy if transplanted), Patient B is older with other health issues (lower life expectancy even with a transplant).

Utilitarian calculus (immediate): Transplanting A might yield, say, 20 QALYs, transplanting B yields 10 QALYs; so pure utilitarianism chooses A for maximising health outcomes.

Prioritarian view: B is worse-off (perhaps currently very ill or with fewer years left), so even though the benefit is smaller in absolute terms, helping B might have greater moral weight – a strict prioritarian might lean towards B *if* the difference in outcome is not too large.

Refined utilitarianism: What rule should govern transplant allocation? One could be maximise QALYs gained per organ (favouring A); another could be giving an equal chance or some priority to older/weaker patients. A refined utilitarian weighs not only the total life-years but also public values. If organs always go to the youngest and fittest, older or disabled patients will feel devalued, and society may view the system as ageist or survival of the fittest, undermining solidarity. On the other hand, if organs always go to the sickest irrespective of outcome, many organs might be effectively wasted, and overall survival declines, which also upsets people who value efficient use of donations. The solution might be a mixed rule: for example, use medical criteria that include urgency of need and probability of benefit, perhaps also a waiting time element (so everyone has some chance). Such a rule might in effect give moderate priority to the worse-off while still considering outcome, a balance that the public often supports [31].

Refined utilitarianism supports this balance because it maximises societal welfare over time; people will donate organs only if they trust the allocation is fair, and patients will accept the process if they know both need and outcome are considered (overall, more lives saved and public contentment). Notice how this reasoning justifies giving some weight to the worse-off (similar to prioritarianism) but ultimately hinges on maximising the success and credibility of the transplant system (a utilitarian objective). Indeed, refined utilitarianism can be seen as utilitarianism with a human face. It acknowledges that things like fairness perceptions, rule of law, and community values are themselves significant contributors to human well-being [32]. In healthcare, a system that coldly maximises QALYs without regard to equity might achieve a bit more health on paper but could incite so much public backlash or distress among patients and providers that overall welfare (in a broad sense) is actually lower. As long as respecting certain egalitarian norms brings about the maximum long-term utility, refined utilitarianism is content to respect those norms (even when, in isolated instances, doing so means not choosing the option with the highest immediate QALYs). Importantly, “acceptance” here is constrained: approval won by misinformation or coercion does not count since refined utilitarianism demands *deliberative* acceptance. This is because rules under refined utilitarianism must be publicly defensible under transparent, non‑manipulative, and well‑informed deliberation. In practice, this favours institutional guardrails which tend to increase welfare by sustaining trust while insulating acceptance from manipulation.

Having defined refined utilitarianism, in the remainder of the article I shall apply this framework to healthcare. One will explore what a purely utilitarian approach to healthcare distribution looks like, and then examine how the current NHS operates, showing that it largely aligns with refined utilitarian reasoning. It will become evident that UK healthcare already implements a combination of utilitarian and egalitarian measures: for routine, abundant care it leans toward egalitarian rules (which refined utilitarianism approves of due to their long-term benefits to social welfare), and for scarce, critical resources it justifiably shifts toward outcome-maximising (utilitarian) strategies. What is more, sections that follow will also identify concrete discriminators (e.g. aggregate‑outcome objective, rule‑level overrides in crisis, consequence‑selected rules with fairness tie‑breakers) in NHS policy to make the refined utilitarian interpretation testable rather than merely programmatic.

## A utilitarian healthcare system: distribution by maximum benefit

If a healthcare system were explicitly utilitarian, its guiding aim would be to maximise health outcomes and overall welfare per resources expended. In practice, this means resources (e.g. money, staff time, organs, medicines, etc.) would be allocated to whatever uses produce the greatest improvement in population health and well-being. The UK’s National Health Service, though founded on egalitarian principles, has gradually incorporated many tools of utilitarian reasoning, most prominently, cost-effectiveness analysis in the form of the Quality-Adjusted Life Year (QALY) metric. To understand both the power and limits of a utilitarian approach, one should examine how QALYs and similar measures function in healthcare distribution.

A QALY is a unit that combines length of life and quality of life into a single metric. By convention, 1 QALY equates to one year of life in perfect health. A year in less than perfect health (say, living with chronic pain or disability) counts as less than 1 QALY (perhaps 0.5, if that state is rated as ‘half’ as good as full health), and death is 0 QALYs [33, 34]. QALYs are typically calculated by multiplying the quality of life score (0–1) associated with a health state by the number of years lived in that state. For example, if a treatment would give a patient 5 additional years of life at a quality-of-life weight of 0.8 (where 1.0 = full health), that yields 5 × 0.8 = 4 QALYs gained. Healthcare interventions can then be compared in terms of cost per QALY gained. A treatment that costs £100,000 and yields 4 QALYs has a ratio of £25,000/QALY. Health economists and policymakers often use such ratios to decide which interventions are ‘worth’ funding. The underlying utilitarian logic is clear: given a fixed budget, to maximise total health benefits one should fund the interventions with the lowest cost per QALY (biggest QALY bang for the buck), up to a certain threshold. In England, the National Institute for Health and Care Excellence (NICE) generally considers an intervention cost-effective if it costs between about £20,000 and £30,000 per QALY or less [35, 36]. In fact, since its inception in 1999, NICE has implicitly adopted a threshold range of roughly £20k–£30k per QALY for recommending treatments in the NHS [37]. Treatments below that threshold are usually approved as good value for money, whereas those above may be rejected or only used in special cases. This thresholded use of cost‑effectiveness is a leading indicator of a *utilitarian* institutional design because aggregate outcome maximisation becomes an explicit system objective and a cross‑programme tie‑breaker, not merely a background virtue like “do not waste”.

From a utilitarian standpoint, the QALY system operationalises utility in healthcare. It treats a year of healthy life as a proxy for utility (happiness or preference satisfaction), assuming that health is a critical component of well-being. By maximising QALYs per pound spent, the health service attempts to maximise the total health utility produced with limited resources. For example, if the NHS must choose between funding Treatment X (which provides small improvements to many people’s quality of life) and Treatment Y (which provides a large life-extension to a few people), the QALY framework will favour whichever yields more aggregate QALYs. If Treatment X helps a lot of patients gain 0.1 QALY each at low cost, it might outscore Treatment Y which gives a smaller number of patients, say, 2 QALYs each at very high cost. This method has the advantage of comparability; it provides a common unit to compare very different health programs (heart surgery vs. mental health therapy vs. cancer drugs) on an ostensibly objective scale of benefit. As a result, QALYs are widely used in health technology assessments and resource allocation decisions around the world [38]. The UK’s NHS, through NICE, uses QALY-based analyses to decide which new medications or treatments to cover, aiming to allocate its budget to maximise health gains for the population [35, 39, 40]. Other moral frameworks may use cost‑effectiveness instrumentally; what is distinctive in the NHS case is that cost‑per‑QALY functions as a public, general rule for distributing limited funds across disease areas – precisely the sort of rule‑level orientation that refined utilitarianism expects [41, 42].

In an ideal utilitarian healthcare model, life-years and health improvements would be allocated to maximise total QALYs. This can lead to some seemingly hard choices: for instance, focusing on prevention and younger patients can often produce more QALYs than treating very old or terminally ill patients. A year of life saved in a child yields many more potential future QALYs than a year of life saved in a 90-year-old. Thus, pure utilitarian logic may prioritise a treatment that saves a 20-year-old over, all other things being equal, a treatment that saves an 85-year-old, if one must choose. Similarly, treatments for patients with good chances of recovery will appear more cost-effective (more QALYs) than treatments for patients with multiple co-morbidities that limit their improvement. This logic underlies certain allocation practices. For example, in some organ transplant programs and intensive care triage, factors like age and baseline health are considered because they affect prognosis [43]. During the COVID-19 pandemic, UK guidelines asked clinicians to consider patients’ frailty and likelihood of recovery when allocating scarce critical care beds, effectively a utilitarian criterion aiming to save the most life-years [44]. Early COVID‑19 guidance referred to use of the Clinical Frailty Scale (CFS) in patients over 65 to inform critical‑care decisions; however, NICE clarified that CFS should not be used as a rigid cut‑off, should not be applied to people with stable long‑term disabilities, and should be combined with holistic clinical judgement [45]. This refinement is exactly what refined utilitarianism predicts: retain prognosis‑based rules in crisis *without* blanket exclusion by age or disability and ensure the rule is publicly defensible. While framed as clinical guidance, the ethical underpinning was utilitarian: maximise the benefit from each ICU bed (i.e. prioritise those likely to leave the hospital alive and in decent health). Indeed, the BMA guidance bluntly stated that during pandemic triage, decisions about meeting individual patients’ needs *“will give way to decisions about how to maximise overall benefit”*, a clearly utilitarian directive [1, p. 6].

However, this QALY-maximisation approach raises serious ethical concerns. One prominent criticism is that it can be unfair to people with disabilities or chronic illnesses, the so-called “double jeopardy” problem [46]. Double jeopardy means that those who are already disadvantaged in health are doubly disadvantaged by a utilitarian allocation, first by having a lower quality of life, and second by being given lower priority for treatment because their treatment yields fewer *additional* QALYs. For example, a person with a disability might, even after treatment, have a quality of life score less than 1.0. If two patients receive the same life-extending surgery, the one who ends up with a perfect health state gains more QALYs than the one who remains in a moderate disability state, simply because of the way QALYs are calculated (the latter’s each year counts as, say, 0.7 instead of 1.0). Utilitarian allocation might favour the already healthy patient because the incremental QALYs from treatment are higher. Critics argue this effectively values the lives of disabled or chronically ill persons less, treating some QALYs as worth more depending on who accrues them (ibid.). Such an implication clashes with the intuition, also rooted in the NHS’s ethos, that each life is of equal value [24]. Relatedly, QALY-based policies risk implying some people’s QALYs matter more than others’, which undermines the utilitarian’s own impartiality ideal (everyone counted equally). Furthermore, giving lower priority to those with worse health (due to disability or age) seems to punish them for circumstances beyond their control, violating a sense of justice [10]. In the literature, proponents of QALYs have responded that the QALY system does not intend to discriminate; it focuses on the change in health, not the person’s intrinsic worth [31, 41, 47]. They note that what is being maximised is health improvement, and in principle a disabled patient could receive just as much improvement as a non-disabled patient, so each QALY is counted the same regardless of whose it is [48]. In other words, a QALY is a QALY, and one year of healthy life is valued equally for any person. If fewer QALYs result from treating a particular patient, that is not because they are valued less, but because the treatment can not benefit them as much. This is the utilitarian rationalisation. Nonetheless, the optics and ethics are difficult. It feels at odds with the principle of equal respect, and it may conflict with anti-discrimination laws like the UK Equality Act 2010, which prohibits less favourable treatment on grounds of disability or age [49]. Indeed, NICE had to be cautious. Its COVID-19 guideline initially risked being seen as de facto age and/or disability discrimination because frailty scores correlate with age and disability. NICE quickly clarified that the Clinical Frailty Scale should not be applied to stable long-term disabled people, and that no one should be denied care simply due to age or disability [44, 45, 50]. The guideline was revised to emphasise individualised assessment and the likely benefit from critical care, not blanket cut-offs [45]. In parallel, NICE has introduced a severity modifier (sometimes called a severity premium) that permits higher willingness‑to‑pay where baseline disease burden is high, signalling that social concerns about severity can be incorporated consistently within a population‑health framework [51]. This is characteristic of refined utilitarianism: adjust the rule‑set to secure long‑run welfare gains (including legitimacy and trust) while avoiding rights‑violating shortcuts; triage policies must balance utility and fairness, and outright exclusion of the elderly or disabled is ethically unjustifiable, both from a deontological view and because it would undermine public support for healthcare rationing [5].

Another issue in a straightforward utilitarian system is the fate of minor gains for many vs. major gains for few. Utilitarian calculus might approve spreading resources to yield small benefits to a large number, rather than a life-saving benefit to a small number, if the total QALYs added up that way. For instance, using a fixed budget to fund a vaccination program that adds 0.1 QALY each to 1,000 people (total 100 QALYs) might be preferred over an expensive cancer drug that adds 5 QALYs each to 10 people (total 50 QALYs). Many would agree with that choice as good public health sense. But taken to an extreme, this approach can conflict with the rule of rescue, our strong moral intuition to save identifiable individuals in mortal peril, even at high cost [52]. The NHS has special provisions (like higher cost-effectiveness thresholds for end-of-life treatments) that acknowledge society’s willingness to pay more per QALY for those in immediate danger of dying [35, 53]. This is again where refined utilitarian reasoning diverges from a narrow utility calculation; it recognises the extra utility (in terms of public moral satisfaction and legitimacy of the health system) gained by honouring the rule of rescue. People feel it is worth spending more to save a life at death’s door, and if a health system never did so (because it was strictly maximising QALYs by focusing on easier gains), it would lose public trust and violate deeply held values [54]. Therefore, refined utilitarianism, while still outcome-oriented, *permits acting in a way that does not immediately maximise utility in a single instance, as long as it brings about maximum long-term utility* (e.g. preserving the ‘sense of community’ that we do not abandon people in dire need). The key is that such exceptions are narrow, transparent, and reviewed, so the long‑run population benefit (including legitimacy) remains the system’s anchor.

In short, a purely utilitarian healthcare distribution focuses on efficiency and aggregate benefit. It has given us valuable tools like QALYs and cost-utility analysis that make trade-offs explicit and help direct funds to where they do the most good. The NHS’s use of an explicit cost-per-QALY threshold (around £20–30k/QALY) is a prime example, a utilitarian-inspired criterion to decide priorities so that, as far as possible, every pound spent yields as much health as it can. Beyond any direct health effects, the use of publicly stated thresholds and processes has arguably improved transparency, consistency, and reason‑giving in NHS decisions – properties that themselves sustain cooperation and trust – which has also helped improve overall health outcomes in the UK by avoiding spending on interventions that give very little benefit relative to cost [55]. However, such a system must be tempered by ethical side-constraints and public values to be acceptable and truly promote well-being in the broad sense. Refined utilitarianism steps in to temper the utilitarian calculus with considerations of fairness, rights, and public trust, not by abandoning utility, but by incorporating those considerations into what *maximising welfare means on a societal scale*. Crucially, “acceptance” for refined utilitarianism is deliberative acceptance: endorsement under transparent, non‑manipulative, well‑informed processes – hence the importance of independent watchdogs, equality impact checks, and public consultation in bodies like NICE (see Table 1). The next section examines how these dynamics play out in the NHS’s actual policies, which mix egalitarian and utilitarian elements.

## Rules and resources: the role of institutions and public values

In practice, healthcare allocation is not decided by running a utility calculation on each patient at the bedside. Instead, institutions and rules guide how resources are distributed. The NHS operates within a framework of laws, guidelines, and ethical codes that shape clinicians’ decisions [30]. This institutional structure can be seen as the embodiment of rules that reflect particular moral principles. A refined utilitarian analysis pays close attention to these rules, how they are formulated, and by whom, because it is often at the rule-making level (policy level) that utilitarian and egalitarian considerations are balanced. On the refined utilitarian view, two architecture‑level commitments do most of the work: (i) rule‑selection is tethered to an explicit population‑outcome objective under conditions of deliberative (transparent, non‑manipulative, well‑informed) public acceptance, and (ii) a rights‑protecting baseline – rooted in equality and consent law – constrains how far outcome‑maximisation may go. In addition, what marks a distinctively utilitarian institutional structure (as opposed to a generic aversion to waste) is that an explicit aggregate‑outcome objective (e.g. cost‑per‑QALY thresholds), rule‑level provisions that allow outcome reasons to override some deontic constraints in specified contexts (e.g. crisis triage), and the use of general rules selected for their long‑run consequences, with fairness mechanisms (e.g. lotteries as tie‑breakers for equal claims) to sustain legitimacy.

In the UK, key rules and principles governing healthcare include:

- The NHS Constitution [24], which enshrines values like universal access, equity, and respect for human rights. It declares that the NHS provides a comprehensive service available to all, with access based on clinical need, not the ability to pay, and that ‘everyone counts’ (ibid.). These statements echo egalitarian ideals and a commitment to equal respect. They set a principle that the NHS should not discriminate unlawfully and should strive to reduce health inequalities. On the refined utilitarian account, such constitutional guarantees function as part of a rights‑protecting baseline that is categorical at system level, because dismantling them would predictably erode trust and thereby depress welfare.
- The Equality Act of 2010, a law that makes it illegal for NHS providers (and others) to discriminate on grounds of protected characteristics, including disability and age. This means, for example, that a hospital cannot have a policy denying treatment to anyone over 75 or to people with disabilities as such. Any differentiation must be objectively justified and based on clinical factors rather than stereotypes or prejudice. This legal backdrop reinforces rules of non-discrimination that the NHS must follow in everyday practice. In refined utilitarian terms, the statute *institutes* a constraint: any outcome‑oriented rule must pass transparent, reason‑giving non‑discrimination tests if it is to be publicly acceptable (see Table 1).
- Professional ethical guidelines (such as the General Medical Council’s codes, or guidelines by medical Royal Colleges) which require doctors to treat patients fairly and without bias, to obtain consent, to prioritise patient welfare, etc. For example, the Royal College of Physicians advises on how to prioritize patients ethically when services are overstretched, emphasising transparency, consistency, and the involvement of clinical judgment rather than arbitrary decisions [56]. Such guidance operationalises fairness requirements (and, where appropriate, equal‑claim tie‑breakers) and embeds a duty to give reasons – features that support legitimacy and thus long‑run welfare.
- Resource allocation bodies like NICE, which issue rules in the form of guidelines for when treatments are approved. NICE’s decisions effectively set upper-level rules about what the NHS will fund. For instance, NICE might stipulate that a certain drug is approved only for patients who meet specific clinical criteria (ensuring the drug is used where it is most effective). These rules are grounded partly in utilitarian reasoning (such as maximise health gain from NHS funds) but also consider equity (eg special funding for rare diseases through ‘Highly Specialised Technologies’ evaluation, acknowledging a societal desire to give some weight to rarity or severity even if cost per QALY is high). Crucially, NICE’s processes (e.g. public consultation, lay membership on committees, published rationales and an appeal route) instantiate the *deliberative‑acceptance* condition central to refined utilitarianism.
- Local policies by Clinical Commissioning Groups (now Integrated Care Boards) or hospital trusts on things like IVF eligibility, elective surgery thresholds, etc. For example, some regions set an age cut-off for NHS-funded IVF (often around age 42), or require patients to have a certain level of obesity reduction or smoking cessation before elective surgery. These are rules often justified by clinical effectiveness and resource constraints, implicitly utilitarian, but they are implemented as blanket policies to avoid case-by-case bias and to appear fair by applying the same rule to all [39, 57].

The existence of such rules and institutional decision-makers reflects a reality emphasised by refined utilitarianism: leaving allocation entirely to individual doctors’ discretion (ie bedside rationing) is problematic. It can lead to inconsistency, hidden biases, and moral distress for clinicians [58]. Instead, having ex ante rules set by authorities can improve overall outcomes and fairness. When the BMA in 2020 recommended that doctors not make certain agonising decisions alone but follow national triage protocols, this was precisely to ensure consistency and to protect clinicians from undue blame [1]. It is generally more acceptable that a decision comes ‘from the system’ with democratic and expert input, than from one harried doctor deciding who lives or dies on the spot. From a refined utilitarian view, centralised rules can reduce bedside rationing and thereby improve trust and reduce moral error (because an individual doctor might be swayed by emotion or bias, whereas a rule is more impartial). For example, during COVID-19, instead of each ICU doctor improvising their own triage criteria (which could vary wildly), the existence of a NICE guideline and hospital policies gave a common foundation. This coordinated approach helped ensure consistent use of scarce capacity and a publicly defensible perception of fairness which are both central to sustaining cooperation and trust.

Another aspect of rules is that they allow transparency and accountability. A purely utilitarian calculation done in someone’s head is opaque and hard to challenge. But a public rule (say, “first come, first served except when emergency cases get priority”) is visible and can be debated. Democratic deliberation can refine such rules to better align with societal values. This is essentially what NICE does by involving lay members and consulting the public on draft guidelines. The refined utilitarian approach favours this inclusive process; if the public is involved, the resulting rules have greater legitimacy and people are more likely to accept outcomes even when unfavourable to them, which in turn contributes to social utility (people remain cooperative and trusting of the system). This is the practical face of refined utilitarianism’s *deliberative‑acceptance* requirement: rules should be ones citizens could endorse under transparent, non‑manipulative, well‑informed conditions.

To connect this to philosophical critiques, one might say that refined utilitarianism at the rule level is incorporating a contractualist element; it seeks rules that no one (or not many) would reasonably reject, because those are the rules that will function well and have broad support [8]. It is not that refined utilitarianism has ceased to be consequentialist, but it recognises that *the* best consequences ensue when moral rules resonate with the public’s sense of justice. Echoing Smart & Williams, one could say refined utilitarianism tries not to legislate to the moral sentiments without their consent; instead it attempts to harness moral sentiments (like compassion for the worst-off, outrage at unfairness) as part of the calculation of overall welfare [11]. The convergence here is justificatory, not motivational; refined utilitarianism still grounds rules in consequences, but it *selects* rules that would survive reasonable rejection because such stability itself increases welfare.

In concrete terms, consider how the NHS handles waiting lists and appointment scheduling. Generally, patients are seen on a first-come, first-served basis modulated by urgency of need. That is a rule. It reflects egalitarian fairness (queuing in order) and prioritarianism (sicker patients jump the queue because they need urgent care). This rule is not necessarily the most utilitarian in each instance. Sometimes, purely in terms of health gain, it might be “efficient” to treat a less sick person first (eg a simple case that can be quickly cured) and leave a complex case for later [59]. But the NHS does not allow queue-jumping based on maximising throughput; it sticks to need-based prioritisation and queue order. Why? Because a *consistent rule of priority by urgency, then by waiting time* is seen as fairest and maintains public trust in the system (everyone feels they will get their turn and the sickest will not be neglected). This yields overall benefits. People are more willing to seek care when needed, comply with referrals, etc, knowing the system is not biased against them. In refined utilitarian terms, the utility of maintaining a sense of community and fairness outweighs any small inefficiencies in not strictly maximising QALYs with each scheduling decision. The NHS’s official principle that patients with the same clinical need should have equal access to care, regardless of personal characteristics or location, serves that long-term utility of equity [24]. Where equal claims truly tie, refined utilitarianism supports impartial tie‑breakers (including lotteries) precisely because they preserve legitimacy without material loss of health benefit (see Table 1).

It is instructive to examine a case where NHS rules incorporate utilitarian logic: organ transplantation. Organs are extremely scarce resources. UK policy for kidney allocation, for instance, uses a point system that factors in waiting time (fairness), tissue match (medical utility for graft survival), age difference between donor and recipient (to maximize graft life-years), and other factors [60]. There is a tilt towards younger recipients in some organ allocation algorithms, because giving a young person a donor kidney can yield decades of life, whereas an older recipient might gain fewer years – a utilitarian consideration. However, it is done in a constrained way and balanced with fairness (all clinically eligible candidates may be listed although eligibility criteria and contraindications apply). Notably, in 2019 the UK Parliament passed the Organ Donation (Deemed Consent) Act 2019, switching to an opt-out system to increase the donor pool. This change was justified on utilitarian grounds (saving more lives), but public acceptance hinged on ensuring safeguards and respecting certain opt-outs [61]. The roll-out involved extensive public awareness campaigns to maintain trust. A purely utilitarian calculus might have no problem with presumed consent since it clearly increases organ supply, but the refined approach ensured the policy was implemented in a way that the public would accept (because if public trust in organ donation collapsed, the utilitarian gain would be lost). Early evidence suggests the opt-out system, combined with respecting families’ wishes to some extent, has indeed improved organ availability without public backlash [60, 62]. This illustrates the refined utilitarian template of combining outcome maximising rules with visible safeguards so that acceptance is earned and not engineered.

From these examples, we see that the NHS’s pattern of rules often follows a mixed ethic: treat people equally and by need (an egalitarian rule), but *within* that, allocate resources to maximise benefit (a utilitarian sub-rule). This aligns with the structure of refined utilitarianism, which can be thought of as a *two-tier system*:

1. **First tier (basic principle)**: Provide care to all who need it, without exclusion – this ensures public buy-in and an equitable baseline (this fulfils deontological and egalitarian constraints that society demands, and contributes to overall welfare by fostering social solidarity and ensuring widespread health coverage). This first tier is part of the rights‑protecting baseline and is not up for suspension in pursuit of marginal gains.
2. **Second tier (priority rules):** When resources are limited, prioritise in a way that maximises health outcomes – e.g. triage by urgency or likelihood of benefit, use cost-effectiveness to choose treatments, etc. – but only in ways that are consistent with the first tier rules or exceptions explicitly built into them. Exceptions should be *narrow, transparent*, and *subject to review*, so that long‑run population benefit (including legitimacy) remains the anchor.

The COVID-19 pandemic really stress-tested this structure. In normal times, the NHS rarely openly prioritises one life over another in a life-or-death scenario; the assumption is there is capacity for all urgent cases (one ICU bed for every patient who truly needs ICU). During the pandemic, that assumption broke down in some places. The BMA and NICE guidelines essentially gave extraordinary rules for an extraordinary context (as allowed by refined utilitarianism) [43, 45]. They maintained as much fairness as possible – for example, NICE’s triage algorithm did not exclude all elderly people but used frailty and prognosis rather than an age cutoff specifically to comply with the spirit of equality law (no one is denied ICU *because* they are 80, but because their assessed chance of recovery is too low) [1, 45]. This nuance was crucial for legal and ethical acceptability [5]. BMA’s guidance also insisted that even when acting utilitarianly, doctors must *“maintain respect for all as ends in themselves”* [1, p. 4], a Kantian phrase, meaning patients should still be treated as individuals, given information, and not treated cruelly. This was a reminder that even under utilitarian rules, the manner of treating people matters (Dworkin would approve of the sentiment at least). The result was a triage policy that, while harsh in outcome for some (those denied scarce ICU beds), strove to uphold transparency, consistency, and compassion [50]. A study of international COVID triage guidelines noted a broad consensus on utilitarian principles combined with safeguards for equal respect [25]. As refined utilitarianism would predict, the guidelines that gained widest acceptance were those that did not simply maximise utilitarian outcomes at any moral cost, but rather incorporated public and professional values (such as giving healthcare workers some priority for their instrumental value and reciprocity, or using lotteries as tie-breakers to preserve equality among similarly situated patients) [63]. Importantly, crisis‑specific rules predated COVID‑19 (e.g. major‑incident triage protocols); the pandemic mainly exposed and extended this crisis code rather than inaugurating an entirely new ethic – supporting the claim that COVID‑19 was a stress‑test of a two‑tier structure already implicit in the NHS.

In short, the creation and enforcement of rules in healthcare are where the spirit of refined utilitarianism is most evident. The NHS’s rules are often chosen because they lead to good outcomes *and* resonate with shared moral norms. Those rules, once in place, ensure that individual decisions by clinicians align with a consistent ethical approach, rather than the system relying on each clinician to perform implicit utilitarian calculations on the fly. A frontline doctor, for example, triages patients in A&E by clinical urgency (per NHS protocol) – that inherently saves the most lives (utilitarian) *and* is seen as fair (no one disagrees that the person bleeding to death should be seen before someone with a sprained ankle). Meanwhile, at higher levels, NICE decides which drugs to fund using QALYs (utilitarian) *but also* sometimes makes exceptions (eg higher threshold for end-of-life treatments, or specific consideration for very rare diseases) acknowledging public values that not all QALYs are equal in the public’s eyes [35, 40]. Those exceptions are exactly where egalitarian or prioritarian intuitions have influenced policy, and refined utilitarianism can rationalise them by the need for societal acceptance of the health system’s priorities. In the next section, I use the above *discriminators* to test whether specific NHS domains (routine services, specialised treatments, crisis triage) instantiate a *distinctively utilitarian* structure constrained by a rights‑protecting baseline and secured through deliberative acceptance.

## The NHS in practice: balancing utilitarian and egalitarian approaches

In what follows I ‘test’ three distinctively utilitarian discriminators against NHS practice: (1) an explicit aggregate‑outcome objective (e.g. cost‑per‑QALY thresholds), (2) rule‑level provisions allowing outcome‑reasons to override some constraints in specified crisis contexts, and (3) selection of general rules for their long‑run consequences with fairness mechanisms (e.g. lotteries for equal claims) to sustain legitimacy.^4^

### Egalitarian distributions in normal conditions

The National Health Service was founded on egalitarian principles of fairness and universalism. Aneurin Bevan, the NHS’s architect, famously stated that healthcare should be provided on the basis of need, not ability to pay, and that everyone, rich or poor, should have equal access [64]. This ethos remains central to the NHS [24]. In day-to-day operations when resources are not acutely scarce, the NHS strives to distribute healthcare without discriminating among patients. Clinical need and waiting time are the main determinants of who gets treated first, not judgments of social worth or potential utility. This looks like a non-utilitarian, egalitarian approach since each person’s life is treated with equal concern, and no one is denied treatment because their expected outcome is worse or because treating someone else would produce more QALYs. On the refined utilitarian view, these everyday rules form part of a *rights‑protecting baseline* that is retained because its public acceptance and fairness effects raise long‑run welfare; they are not mere window‑dressing.

For example, if two patients arrive at an A&E department, both needing urgent care but one has slightly better odds of recovery, the doctors will not (and ethically must not) send away the patient with poorer odds in favour of the other. They will treat both to the best of their ability, starting with whichever is more critically ill (the most urgent). This reflects what philosophers call a commitment to equal respect and the intrinsic value of each life [32]. As the NHS Constitution puts it, *“everyone counts”*, and *“the NHS has a responsibility to provide fair and equitable services”* [24]. In ethical terms, this aligns with deontological and egalitarian theories that stress fairness and the protection of individuals. For instance, philosopher John [65], famously argued that when faced with a choice of saving one person or five, one should not automatically prefer the five; instead, one could flip a coin to give each person an equal chance, because numbers *per se* might not trump the separateness and worth of individuals. Taurek’s provocative stance encapsulates the egalitarian intuition for equal chances. In practical healthcare, while we do not literally randomise in every situation (except perhaps as a tie-breaker), the NHS does implement similar fairness ideas. When multiple patients have equivalent need and prognosis, they should have an equal chance to receive treatment (often achieved by first-come-first-served or lottery if needed). This is seen, for example, in organ allocation tie-breaks or in how access to experimental treatments via lotteries has been occasionally used in extreme scarcity [66]. Where equal claims truly tie, refined utilitarianism *endorses* impartial tie‑breakers precisely because they preserve perceived fairness without sacrificing material health benefit, thereby supporting trust over time.

Beyond immediate triage, the NHS also has policies to prevent discrimination against groups who historically faced bias. The Equality Act of 2010 makes it unlawful to deny or dilute care based on protected characteristics like race, gender, age (with some qualified exceptions), or disability. This has concrete implications. For example, a General Practice cannot refuse to register an elderly or disabled patient because they might need more care. Hospitals cannot have a blanket policy of not offering surgery to people above a certain age (unless clinically justified by individual assessment), doing so would be age discrimination. Similarly, NHS guidelines affirm that treatment decisions should be made on clinical criteria, not on social value judgments about patients’ worth [1, 35, 36]. In routine practice, patient autonomy and rights are respected; patients cannot be forced to do what yields the most utility (for example, a patient can decline a treatment even if it is cost-effective, and doctors must respect that choice due to the ethical principle of consent). This respect for autonomy and non-discrimination aligns with liberal egalitarian theories (like Ronald Dworkin’s view that each person must be treated with equal concern and respect by public institutions) [9]. It also resonates with Kantian ethics, treating individuals as ends in themselves, never merely as means to an end, which is explicitly echoed in NHS values and the BMA’s emphasis on maintaining respect for persons even in utilitarian triage [67]. From the perspective of refined utilitarianism, these legal‑ethical constraints are *constitutive* of a publicly acceptable code; dismantling them would predictably erode trust, reduce care‑seeking, and thereby lower population welfare.

However, the NHS’s egalitarian practices operate within certain practical limits. When healthcare resources are sufficient to meet demand (or almost sufficient), egalitarian distribution is relatively easy to uphold – everyone who needs treatment gets it, and nobody is excluded. But as demand comes to exceed supply, even in less acute ways, the NHS must adopt some priority criteria. Typically, it continues to use need or severity as the criterion [51]. This is inherently a form of prioritarianism (worse-off patients get treated first) in everyday healthcare. For instance, patients with more serious illnesses are put at the top of waiting lists [44]. That is to say, if two people need a hospital bed and one’s condition is life-threatening while the other’s is stable, the life-threatening case gets the bed. This can be framed as utilitarian (saving the life yields more benefit than treating a stable case) or as prioritarian (the sickest deserve help first). In the language of refined utilitarianism, urgency is in line with the prioritarian view and by prioritising urgency, the NHS follows a rule that society overwhelmingly supports and that indeed tends to maximise overall health outcomes [68]. Crucially, this is rule‑level design, not bedside maximisation: a general priority rule for routine conditions that is publicly knowable and broadly endorsed.

Consider elective surgery scheduling. The NHS has referral-to-treatment time targets that usually range from 18 weeks for non-urgent procedures, but urgent cancer surgeries have a target of 2 weeks from suspicion to specialist consult and 31 days from decision to treat to treatment [69]. This means cancer (life-threatening) is fast-tracked, whereas a non-urgent hernia repair might wait longer. Again, severity/urgency trumps other considerations. This reflects both a moral commitment to not let people suffer or die when one could prevent it, and a utilitarian rationale that preventing a death or serious deterioration yields a larger utility gain than fixing a minor issue. The public articulation of these targets also satisfies refined utilitarianism’s *deliberative‑acceptance* condition (i.e. transparent, reason‑giving rules that can be endorsed even by those awaiting less urgent care).

One might ask: does the NHS *ever* treat someone with less need before someone with greater need? Generally no, not intentionally. But sometimes blanket egalitarian policies can lead to paradoxes that utilitarian reasoning seeks to address. A classic debate in medical ethics is first-come, first-served vs. outcome-based triage [70, 71]. First-come-first-served is intuitively fair (and is how NHS waiting lists largely function), but it can lead to suboptimal outcomes if, say, someone who arrives slightly later could have been saved while the one who arrived just before them will die despite treatment [70]. In emergencies, the NHS deviates from strict queue order, as that is what triage is [25]. In a multi-casualty incident, a triage officer might actually delay care for an extremely injured (likely unsalvageable) victim in order to treat several moderately injured who can be saved. This is a more utilitarian approach justified in extreme scenarios. However, the general triage protocol still has an egalitarian facet; everyone is assessed quickly, and categories are made (immediate, delayed, minimal, expectant) based on chances of benefit [5]. The expectant category (those not likely to survive) receives palliative care rather than aggressive treatment in a mass casualty situation – a stark utilitarian necessity. Outside such dire scenarios, the NHS does not use an expectant category. All patients are given active care unless it is truly futile for that individual (as determined by clinical judgment, not because someone else could benefit more) [72]. . These protocols exemplify a stable *two‑tier* code (routine vs. crisis) rather than an ad‑hoc swing to maximisation.

Another egalitarian element in normal NHS operations is the use of lotteries or equal splits in certain dilemmas. For instance, during the early COVID vaccine rollout, the UK followed a prioritarian scheme (elderly and vulnerable first), but within a given priority group, essentially it was random or first-come (people booked appointments, etc, but there was no further fine triaging by health status once broad groups were set) [73]. If doses were extremely limited and two identical individuals wanted the last dose, a lottery might be a fair solution. Ethically, lotteries are sometimes recommended to preserve equality when candidates have equal claims [63, 74]. The NHS Blood and Transplant service also occasionally uses a random allocation if two patients are virtually tied on points for an organ offer [62]. These methods demonstrate the commitment to giving each person a fair chance, rather than squeezing out a few more QALYs by picking, say, the slightly younger or more deserving in a subjective sense. This is exactly the sort of fairness mechanism refined utilitarianism favours to protect legitimacy where outcome differences are negligible.

Equality of access also means NHS treatments are (in theory) distributed without regard to wealth or status. A millionaire and an unemployed person get the same treatment on the NHS [64]. This is deeply egalitarian and a point of national pride. It does carry utilitarian benefits (a healthier population overall, less spread of disease, etc), but primarily it is a justice principle. Refined utilitarianism would say the social solidarity and absence of resentment from providing equal access contribute greatly to social welfare; a healthcare system perceived as two-tier and unfair can cause social conflict and diminish trust (which is detrimental to well-being) [75]. Thus, even from a broadly utilitarian perspective, maintaining universal healthcare increases overall welfare by ensuring everyone seeks care when needed (improving public health) and by reinforcing social cohesion (people value living in a society where everyone is cared for). Framed this way, equal access is not a concession *to* utilitarianism but a *component* of the welfare‑maximising public code.

Yet, the NHS’s egalitarianism is not absolute. There are implicit ways in which utilitarian considerations creep in, especially as one moves from clinical triage (micro level) to health policy (macro level). The next subsection will look at how the NHS’s seemingly egalitarian system overlaps with utilitarian logic when making tough decisions, revealing the *verdict* that UK healthcare, in practice, follows refined utilitarianism.

## The Overlap and the Verdict: A Refined Utilitarian System

On close examination, UK healthcare is neither purely utilitarian nor purely egalitarian, it is a hybrid that can be best understood through refined utilitarianism. In day-to-day abundant conditions, it behaves largely on egalitarian (or prioritarian) basis, whereas in situations of scarcity or high stakes trade-offs, utilitarian reasoning becomes more pronounced. Crucially, these shifts happen in a way that still seeks public legitimacy. The refined utilitarian thesis would predict exactly this pattern: general rules that honour equality and need, combined with special policies to maximise outcomes when necessary, all under an umbrella of public acceptance.

Let us survey specific practices and policies to see this interplay:

- **Routine care (GP visits, A&E, inpatient care):** As described, allocation is by need and queue order. No one is excluded. This can be seen as the system operating under a rule: *“Always treat patients in order of clinical need (urgency/severity)”.* This rule is compatible with utilitarianism because, as noted, it often maximises health benefits, treating urgent cases first prevents deaths and complications, which is efficient in outcome terms. And treating less urgent later does not usually cause much loss except some discomfort waiting. The rule also has nearly universal public support; any deviation (like treating a minor case before a critical case) would cause outrage and clearly reduce trust [41, 52]. So refined utilitarianism endorses the need-based rule strongly. This is a general, publicly knowable rule – and not bedside numerics – hence fits refined utilitarianism’s institutional focus.
- **Preventive and public health services:** The NHS provides immunisations, screenings, and public health interventions broadly equally (everyone gets an invite for bowel cancer screening at 50, for example) [76]. This equal opportunity is fair and also maximises uptake which maximises overall health benefit. One might note certain targeted programs (eg more resources to areas with worse health outcomes) – that is a prioritarian tweak (called *“proportionate universalism”* in public health) [77]. Again, done for both equity and overall benefit (improving worst-off yields big health gains). For instance, physical activity programs for disabled adults invest in a worse-off group to improve their health, which reduces health inequalities and increases total wellness, a win-win [78]. Refined utilitarianism explains this convergence instrumentally: priority to the worse‑off is justified where it predictably increases long‑run welfare and acceptance (see Table 1).
- **Specialised treatments and “rationing”:** Here is where the NHS must confront limits more explicitly. Examples include:
  - **IVF fertility treatment:** NHS offers a limited number of IVF cycles to women up to a certain age (typically up to age 42 under NICE guidelines, but many local commissioners cap at 35–39 due to resource constraints) [39, 57]. Denying IVF to older women is controversial, as it seems ageist. The justification is utilitarian: success rates drop sharply with age, so beyond a certain age, IVF on the NHS has low yield and high cost per successful birth. However, to make the rule more palatable, it is framed as an clinical effectiveness cutoff rather than “your life is worth less”. The rule is set openly (public knows the cutoff) and is based on data [79]. This is a refined utilitarian compromise – a blanket rule (so it is fair in application) that is grounded in outcome maximisation (younger patients first because they benefit more). The controversy around this (women in their late 30s feeling discriminated) illustrates the tension. But note, the rule is not absolute nationally. Variation exists, which has been critiqued as a “postcode lottery” (ie unfair) [80]. NICE recommends up to age 42 offering IVF, implicitly saying that beyond 42 the utility drops so much that it is not recommended [39]. That is a prioritarian/utilitarian judgment balanced, since they extended it to 42 to be somewhat inclusive, even though beyond 40 the success is low, possibly due to a value judgment that giving some chance is worthwhile within that range [40]. Refined utilitarianism expects such openly debated, data‑based cut‑offs, constrained by equality law and subject to periodic review.
  - **Organ transplants:** As discussed, point systems include outcome-based criteria. Yet all clinically eligible candidates may be listed (eligibility criteria and contraindications apply). No one is told “you cannot get a liver because someone else would get more years out of it” in those words, though the effect might be similar if the algorithm heavily favours younger recipients. The refined approach here is to incorporate utilitarian factors subtly (through scoring) while maintaining that all patients are treated under a uniform system. And indeed, that system is periodically reviewed with public input, often adjusting to ensure it is seen as fair (e.g. giving some priority to those who have waited longer). This mix – utility‑sensitive scoring plus fairness constraints and public review – matches refined utilitarianism’s template.
  - **Expensive medications:** NICE’s technology appraisals sometimes withhold very costly drugs that provide modest benefit. For instance, a cancer drug that extends life by 2 months at a cost of £100,000 might not be approved. This is plainly utilitarian rationing (not spending a lot for little gain so the money can go elsewhere for more gain). However, NICE has carve-outs: End-of-Life criteria allow approving drugs that exceed the normal cost/QALY threshold if they are for terminal illnesses with small patient populations [35]. Why? Because society feels a special obligation to try to extend life for those in desperate straits (the rule of rescue in another guise). So, NICE will pay more per QALY for a drug that adds, say, 3 months of life for a patient with advanced cancer, than it would pay per QALY for a routine treatment. This is explicitly to reflect social values of compassion even when not strictly cost-effective [53]. Refined utilitarianism can justify this by the idea that the public’s *utility function* is not linear in QALYs. People derive a lot of moral satisfaction or peace of mind knowing the NHS will not abandon the dying, which is a kind of welfare benefit unaccounted for in QALY numbers [81]. Thus the *overall* utility might be higher if such patients are given some hope, even at higher cost. Crucially, these exceptions are narrow, transparent, and revisable, so the system’s anchor remains a population‑health objective.
  - **“No treatment” decisions:** Doctors sometimes decide not to offer treatments that have very low chance of benefit (clinical futility). This can be seen as utilitarian (as it does not waste resources) but is also standard medical ethics (do no harm, do not give false hope, etc.). The Liverpool Care Pathway controversy a decade ago (guidelines for end-of-life care) showed how delicate this is – if people think doctors are denying care for utilitarian reasons, backlash ensues [82]. The solution was to emphasise individualised compassionate care planning rather than any blanket pathway that could be misinterpreted as rationing (ibid.). So refined utilitarianism would caution that even if it is rational to withdraw futile care, it must be handled transparently and with family involvement, or trust in the system erodes (reducing overall welfare). Again, the point is institutional design; outcomes matter, but *how* outcomes are pursued is part of welfare.
- **Pandemic triage:** We have already covered that in COVID-19, rules shifted to open utilitarian prioritisation (eg frailty and prognosis criteria, potential withdrawal of ventilators for redistribution). The public, though anxious, largely accepted this as necessary given the framing that *everyone is treated equal in worth, but to save more lives we must make hard choices* [63, 83]. It helped that authoritative bodies such as NICE and BMA took responsibility for these rules. And indeed, surveys during COVID found strong support for prioritising those likelier to survive and frontline workers, etc, which aligns with utilitarian logic tempered by reciprocity [5, 84]. Importantly, once the acute crisis passed, those emergency rules were stood down, demonstrating context contingency. More broadly, crisis‑specific rules pre‑dated COVID (e.g. major‑incident triage); the pandemic *exposed and extended* an existing crisis code. This supports the view that COVID‑19 operated as a stress‑test of a two‑tier structure already implicit in the NHS rather than a wholesale ethical shift.
- **Healthcare workers priority:** A subtle example of refined utilitarian policy is giving NHS staff priority for certain things for the greater good. During COVID, as noted, NHS and care staff got priority for weekly COVID testing and eventually for vaccines early on [85]. Also initially, there was a time, the very scarce antibody test was offered only to NHS and care staff, not the general public [86]. This is a clear utilitarian move (get staff back to work, reduce spread in hospitals). It technically ‘discriminates’ by occupation, but it was widely accepted as sensible. Refined utilitarianism would likewise aim to be transparent unless transparency itself would cause a backlash that reduces compliance. In this case, it was no secret that health workers were prioritised; it was publicly announced as policy (ibid.) The public clapped for NHS and likely accepted they should get first access to tests because it benefits everyone if nurses are not off sick unnecessarily. So, again a rule with both an ethical narrative (protect those who protect us). Refined utilitarianism explains both the content and the presentation: the *rule* elevates outcomes and is publicly defensible under transparent justification.

All these observations lead to the conclusion: UK healthcare is utilitarian in its outcomes to a large extent, but it achieves this through a system of rules and norms that are also sensitive to fairness and public values. In other words, it is *refined utilitarianism in action*. The covert utilitarianism in seemingly egalitarian policies (like equal access, or giving priority to the sickest) becomes evident when you consider the long-term effect: these policies foster trust, encourage people to seek care early, and maintain social solidarity, all of which contribute to better health outcomes and social welfare. Conversely, where purely egalitarian approaches would seriously undermine efficiency (as in extreme scarcity), the system carefully steps toward utilitarian choices, but does so under the guidance of publicly accountable bodies and with ethical oversight. Read this not as a motivational claim about NHS philosophy, but as a best explanatory fit between observed institutional patterns and the structure of refined utilitarianism (objective, crisis code, fairness‑sustaining rules).

## Conclusion

The COVID-19 pandemic cast a sharp light on the ethical foundations of the NHS, forcing an explicit shift toward utilitarian decision-making in the face of overwhelming scarcity. One began with the BMA’s call for a pandemic ethical balance tilted toward utilitarian objectives. I emphasised that this did *not* inaugurate a new ethic for UK healthcare; rather, the pandemic *stress‑tested* a crisis code that was already implicit in institutional guidance (major‑incident triage, prognosis‑sensitive allocation). This prompted the question of whether the NHS in normal times is fundamentally utilitarian or not. Through our analysis, I have argued that the NHS is *utilitarian in a refined sense*: it seeks to maximise health and well-being across the population, but it does so by adopting rules and practices that respect societal values of fairness, rights, and compassion. Read in this way, NHS practice instantiates what I have called *refined utilitarianism*, identified by three discriminators: an explicit aggregate‑outcome objective at system level (e.g. cost‑per‑QALY thresholds), rule‑level provisions that permit outcome‑based overrides in specified crisis contexts, and general rules chosen because of their long‑run consequences, paired with fairness mechanisms (e.g. lotteries for equal claims) to sustain legitimacy (see Table 1). In effect, the NHS exemplifies refined utilitarianism, a middle path that incorporates the strengths of utilitarian efficiency and egalitarian justice.

How does refined utilitarianism differ from rule utilitarianism and prioritarianism? I can now answer clearly:

- Unlike *classical rule utilitarianism*, refined utilitarianism does not require a rigid, one-size-fits-all rule set derived purely from utility calculations. Instead, it allows for context-dependent rules and actively considers public attitudes in rule selection. The NHS does not follow a single algorithm to maximise QALYs at all times. It operates with one set of rules (eg treat patients equally and by need) in ordinary situations and shifts to another set (eg triage by prognosis) in emergencies. Classical rule utilitarianism can of course model context‑sensitivity (e.g. via “ideal code” or two‑level accounts), but refined utilitarianism makes this institutional: it builds explicit *context* codes, requires *deliberative* public acceptance (transparent, non‑manipulative, well‑informed), and anchors decision‑making in a *rights‑protecting* baseline (e.g. non‑discrimination, consent). Accordingly, refined utilitarianism can *stably* endorse maintaining a slightly sub‑optimal local rule when changing it would predictably erode trust and thereby reduce overall welfare – something the classical presentation does not foreground.
- Compared to *prioritarianism*, refined utilitarianism shares the intuition that helping the worse-off often deserves priority, but it grounds that in overall welfare rather than in an intrinsic moral weight. The NHS’s many prioritarian-leaning policies, from emergency triage of the sickest first, to focusing resources on deprived areas, achieve outcomes that prioritarian theory endorses (improving the lot of the worst-off, thereby often reducing inequality). However, the justification within the NHS framework is ultimately consequentialist (it improves population health, and fairness is instrumentally good) rather than purely because inequality is bad in itself. We saw that where a strict prioritarian might keep treating a worst-off patient even if it yields very little benefit, the NHS will stop if it is futile (utility = zero). On the refined utilitarian account, priority for the worse‑off is supported where it predictably raises long‑run welfare and public acceptance [e.g. “rule of rescue”, severity‑sensitive thresholds), without elevating priority to a lexical constraint over outcomes. This preserves convergence with prioritarian practice in many cases while keeping the population‑welfare end‑point firmly in view.

Engaging with the primary philosophical sources underscores this point. John Rawls [6], would likely remain critical of any utilitarian foundation for healthcare, arguing that justice requires firm side-constraints. Yet interestingly, the NHS’s guarantee of equal access regardless of wealth and its commitment to protecting the vulnerable do echo Rawlsian commitments – they are there, however, not because utilitarianism ‘found religion’ but because utilitarians realise those commitments are essential for a stable, healthy society (a point even Harsanyi, a utilitarian economist, conceded in debate with Rawls [87]. T. M. Scanlon [8], might still say the NHS’s decisions should be justifiable person-to-person, not just by aggregate benefit. In practice, NHS rationing decisions increasingly involve public reasoning and transparency (consultations, citizens’ juries on tricky questions like “What We Owe to Each Other” in healthcare). This is a convergence where refined utilitarianism and contractualism meet: the NHS tries to find policies that no group finds egregiously unreasonable, because that consensus yields better compliance and outcomes [a pragmatic melding of Scanlonian and utilitarian thinking). Ronald Dworkin [9], who emphasised *rights as trumps*, might view certain NHS practices (like not funding some life-extending drugs] as violating a right to life or equal respect. The NHS would answer that it must balance everyone’s claims – it cannot dedicate unlimited resources to one person without short-changing others, and that in itself would be disrespectful to those others. Dworkin’s ideal of equal concern is arguably present in the NHS’s ethos, as every life is valued, which is why decisions to deny treatment are made reluctantly and with much scrutiny [24]. The refined utilitarian NHS tries to show equal concern *by process*: each patient is given a fair assessment, and none is denied beneficial treatment without strong reason. And when someone must be denied [due to scarcity), it is done under general rules that treat all equally, *never* because that person’s life is deemed less worthy. Bernard Williams, our constant utilitarian sceptic, might grant that the NHS’s approach avoids many of utilitarianism’s pitfalls. It does not ask individual doctors to become utility-maximising machines since they still act on professional duties and compassion. Williams [10], worried about the integrity of individuals under utilitarian dictates. The refined utilitarian NHS largely shields individual clinicians from direct ‘utility vs. integrity’ dilemmas by setting system-level policies. A doctor is not personally deciding to sacrifice Patient B for Patient A; they are following an agreed protocol that society has (hopefully] legitimated. This might not satisfy Williams entirely (the moral residue remains, doctors did feel anguish during COVID triage, for example, as some reports attest [83], but it certainly softens the blow and provides moral support to practitioners (“we did all we could under the circumstances, according to rules developed by our ethical and professional bodies”). Crucially, because refined utilitarianism requires *deliberative* acceptance, it treats manufactured or manipulated consent as normatively invalid; institutional guardrails are part of the theory’s architecture, and not after‑thoughts.

In conclusion, the NHS’s complex moral tissue validates the concept of refined utilitarianism. It demonstrates that utilitarianism *need not be* the callous, numbers-only doctrine its critics portray, if it is refined by a rich understanding of human psychology, social norms, and the demands of justice. Refined utilitarianism in healthcare means pursuing the greatest health benefits while adopting a broad outlook on what welfare entails. It is not just raw QALYs but also peace of mind, public trust, and a sense of fairness. These factors, as I argued, have concrete impacts on health outcomes (for instance, public trust leads to cooperation with vaccination campaigns, affecting community health). Thus, by incorporating social and cultural perspectives into the utility calculus, refined utilitarianism ends up with a system strikingly like the one we have. On this interpretation, the NHS’s egalitarian elements are not counter‑utilitarian anomalies; they *contribute* to long‑run welfare when embedded in a publicly defensible code.

For policymakers and bioethicists, the lesson is that designing a morally defensible and effective health system requires moving beyond simplistic utilitarian or egalitarian formulas. *A blend is needed*. The NHS’s ongoing challenge is to maintain this balance in the face of evolving pressures, such as aging populations, costly new treatments, or pandemics, and shifting public expectations. Refined utilitarianism does not give a once-and-for-all solution, but it provides a method: continuously adjust rules to maximise overall welfare *as defined not only by health metrics but by the values people attach to health and care*. This aligns with the trend of public engagement in NHS priority-setting. It also differentiates refined utilitarianism from a technocratic approach – it must listen to what people consider part of their welfare. Sometimes the public may favour a policy that yields fewer QALYs but more procedural justice (eg, lottery for organ allocation in tie cases), and the refined utilitarian will accept that, because respecting people’s moral preferences is part of maximising their welfare. At the same time, refined utilitarianism’s acceptance condition is normative, not merely sociological: endorsement must be secured under transparent, non‑manipulative, well‑informed processes – so populist drift or manufactured consent do not set the rules.

In the final analysis, UK healthcare can be seen as *neither straightforwardly utilitarian nor egalitarian, but a well-calibrated synthesis*. On balance, the NHS is often characterised as delivering comparatively strong value for money; the NHS manages to achieve admirable health outcomes per expenditure (a kind of macro-level utilitarian success) while retaining broad public support and a reputation for equity. Other health systems might take note. Extreme libertarian or purely utilitarian healthcare models often falter on issues of fairness, whereas purely egalitarian ones can become unsustainable. The refined utilitarian framework offers a promising reconciliation, provided one remembers that numbers matter, but so do norms. In healthcare, perhaps more than any domain, the pursuit of the greatest good must be continuously refined by justice and compassion – a truth the NHS has embodied for over 70 years. A realistic next step is to make explicit, in guidance and public communications, the distinctively utilitarian features already in use (e.g. aggregate‑outcome objective, crisis‑specific rules, fairness‑sustaining tie‑breakers) alongside the rights‑protecting baseline – consolidating legitimacy for the difficult decisions ahead.

## Limitations and directions

This article offers an interpretive, normative diagnosis rather than an empirical causal test of public attitudes and outcomes. Documentary analysis and philosophical argument cannot determine motive or measure welfare directly; they identify the best explanatory fit between institutional rules and ethical theories. Future work should (i) assess how specific fairness mechanisms (e.g. lotteries, severity modifiers) affect trust and uptake, (ii) examine boundary cases (e.g. cancer‑drug funds or rare‑disease exceptions) where public support risks inefficiency, and (iii) explore metrics for “deliberative acceptance” distinct from polls (e.g. reason‑giving quality and equality‑impact checks) so that refined utilitarianism remains non‑ad hoc and non‑manipulable.

## Data Availability

Not applicable.

## Footnotes

2 A note on what is (and is not) distinctively utilitarian: Because deontologists also condemn waste, merely considering outcomes (e.g. using cost‑effectiveness analysis) does not by itself make a policy utilitarian in structure. Refined utilitarianism treats a policy as distinctively utilitarian when: (i) aggregate outcome is an explicit system objective (e.g. cost‑per‑QALY thresholds), (ii) outcome reasons can, under specified conditions, override some deontic constraints at the rule level (e.g. prognosis‑based triage in crisis), and (iii) choices are set by general rules selected for their consequences, with fairness mechanisms (lotteries, equal‑claims tie‑breakers, etc.) calibrated to sustain legitimacy and thus long‑run welfare. These markers guide the assessment of NHS practice below.

3 Parliamentary debates about rescue medicine sometimes echo this sentiment that any chance for the worst-off is invaluable.

4 I, of course, do not claim that NHS actors are motivated by utilitarianism; the claim is interpretive and justificatory: these patterns fit, and are well‑explained by, refined utilitarianism.
